# A new perspective on AMD pathogenesis: a sequential Factor H-centered view of complement dysregulation

**DOI:** 10.3389/fimmu.2026.1814415

**Published:** 2026-04-23

**Authors:** Christine Skerka, Björn Cochlovius, Judith P. Hüllebrand, Deepti Goel, Purnima Sood, Nikhil Pal, Arindam Chakravarti, Daniel R. Muth, Oliver Zeitz, Peter F. Zipfel

**Affiliations:** 1 Koania Complement Analytics, Hamburg, Germany; 2 eleva GmbH, Freiburg, Germany; 3 MM Eyetech Institute, Centre for Sight, New Delhi, India; 4 The Sight Avenue Hospital, New Delhi, India; 5 Aakash Healthcare, New Delhi, India; 6 Division of Eye and Vision, Department of Clinical Neuroscience, Karolinska Institutet, Stockholm, Sweden; 7 St. Erik Eye Hospital, Solna, Sweden; 8 Department of Ophthalmology, University Hospital Zurich, University of Zurich, Zurich, Switzerland; 9 Department of Ophthalmology, Oslo University Hospital, Oslo, Norway; 10 Department of Ophthalmology, Charité - Universitätsmedizin Berlin, Corporate Member of Freie Universität Berlin, Humboldt- Universität zu Berlin and Berlin Institute of Health, Berlin, Germany

**Keywords:** age-related macular degeneration, complement, complement inhibitors, Factor H, Factor H-related protein 1

## Abstract

Age-related macular degeneration (AMD) is a chronic, progressive, retinal disease that primarily affects older individuals and is one of the leading causes of blindness worldwide. Both genetic predisposition and environmental factors contribute to its development. Landmark genome-wide association studies (GWAS) positioned the complement system at the center of AMD research, opening new avenues for understanding disease mechanisms and developing targeted therapies. Among the key complement regulators, Factor H and its splice variant FHL-1, are best known for their roles in inhibiting the alternative pathway. Recent research has expanded our understanding of Factor H, revealing a range of non-canonical functions beyond complement regulation which might also affect AMD pathology. These new functions include roles in cell signaling, tissue protection, metabolism, homeostasis, and modulation of inflammation. In contrast, the related protein FHR1 which is also associated with AMD, exhibits pro-inflammatory properties, promoting monocyte recruitment and activation to facilitate clearance processes. In this review, we summarize the canonical and non-canonical functions of Factor H, FHL-1, and FHR1, and we show how the coordinated action of these three proteins integrates into the broader scope of AMD pathogenesis, including complement activation, inflammation, and photoreceptor degeneration. We also describe the current status of approved complement inhibitors in AMD and emerging therapeutic targets within the complement cascade.

## 1 Introduction

AMD is a multifactorial, progressive, degenerative, and inflammatory retinal disease that primarily affects the elderly. It is the leading cause of blindness in this population and significantly reduces quality of life (1, 2). AMD causes irreversible vision loss through the degeneration of retinal photoreceptors and the retinal pigment epithelium (RPE). Its global impact is escalating: the number of individuals affected by AMD-related vision impairment and blindness rose from 3.64 million in 1990 to over 8.05 million in 2021 (3). Meta-analyses from the Vision Loss Expert Group and the GBD 2019 Blindness and Vision Impairment Collaborators confirm AMD as a major and worsening public health concern (4).

A combination of genetic predisposing factors, age-related cellular changes, and environmental factors drive the development of AMD. Structurally visible hallmarks of early AMD pathology are the formation of drusen and sub retinal drusenoid deposits which are accumulations of lipids, lipoproteins, and proteins located primarily in the sub retinal space between the basal membrane of the RPE and the inner collagenous layer of Bruch’s membrane (BM) (5). These deposits are closely associated with chronic inflammation, oxidative stress, and the progressive loss of RPE cells, which are essential for photoreceptor maintenance. As photoreceptors become irreversibly damaged, they give rise to atrophic lesions in the retina, ultimately leading to loss of visual acuity (6).

This review aims to provide a comprehensive overview of complement mediated immunomodulatory reactions across the stages of dry AMD progression, with the focus on Factor H, FHL-1 and FHR1. First we will discuss the background of the disease and the genetic predisposition which leads to complement dysregulation and the associated para-inflammation. We will introduce the complement extracellular activation pathways and the participating intracellular complement system and develop a model how Factor H, FHL-1 and FHR1 sequentially act in regulating the complement system and influencing inflammation. Subsequently we will review therapeutic approaches to regulate complement activation and provide an extensive summary of latest advancements for prevention and cure of dry AMD.

## 2 AMD

According to the Classification Committee of the Beckman Initiative for Macular Research (7), AMD can be divided into three major stages: Early AMD, which is characterized by the presence of medium-sized drusen (63–125 µm) without pigmentary abnormalities or visual impairment, Intermediate AMD, which is defined by the presence of large drusen (>125 µm) and/or retinal pigmentary abnormalities, often accompanied by mild visual impairment, and Advanced (Late) AMD. The latter represents a stage with two distinct forms, both associated with significant vision loss: Dry AMD which accounts for 80–90% of AMD cases and progresses slowly over time (8). It involves the gradual degeneration of retinal pigment epithelium (RPE) and photoreceptors and eventually results in a complete RPE and outer retinal atrophy (cRORA) usually termed geographic atrophy (GA). Wet AMD (CNV or neovascular AMD) progresses more rapidly and is characterized by macular neovascularization (MNV) which are abnormal newly formed blood vessels that can originate intraretinally (MNV type 3) or through subretinally with growth through the BM (MNV type 1 and type 2 and combinations). The neovascularizations are leaky and lead to intra- and sub retinal edema, hemorrhage, and through that to rapid progression of irreversible central vision loss. Neovascularization is driven by oxidative stress and complement activation, resulting in increased expression of vascular endothelial growth factor (VEGF) (9–11). Both, neovascular and dry AMD, are associated with severe central vision loss, often accompanied by hemorrhages, fibrotic scarring and atrophy.

### 2.1 The role of lipids in AMD

Although the precise sequence of events leading to the development of AMD remains still unclear, it is widely hypothesized that age-related changes, dysregulated lipid and lipoprotein metabolism, and oxidative stress contribute to the formation of drusen and sub retinal drusenoid deposits, followed by chronic inflammation.

Indeed, the link between lipids and AMD has been recognized since the initial characterization of drusen, which are composed largely of extracellular lipid-rich material, including lipoprotein particles containing apolipoproteins ApoB and ApoE (12–14). Lipids, including fatty acids and cholesterol, derived from both dietary sources and photoreceptor outer segments processed by the RPE, are part of a physiological lipid recycling pathway (15). However, in AMD, this balance is disrupted.

Large GWAS have linked AMD not only to complement genes, but also to genes involved in lipid metabolism pathways (16–18). These findings strongly implicate lipids (including those found in drusen) as causative factors in GA. Lipids are a heterogeneous group of molecules, including cholesterol and triglycerides, which are transported into the retina via lipoprotein receptors and also facilitate cholesterol efflux. Under normal conditions, lipoproteins cross the Bruch’s membrane through receptors such as ApoE/A1, ABCA1/G1, and SR-BI/II (19).

In AMD, lipoproteins become trapped within Bruch’s membrane, leading to drusen formation (13–15). This can be followed by oxidative modification, creating activation surfaces for the complement system, which would explain the frequent detection of complement components, including C3b and terminal complement proteins, within drusen (20).

Moreover, the retinal macula is a region characterized by a high density of photoreceptors and intense metabolic activity (21), necessitating substantial blood flow to support its function (1, 15). This high metabolic activity generates large quantities of reactive oxygen species (22), which must be effectively neutralized to maintain cellular health. RPE cells typically manage the clearance of photoreceptor-derived waste products, including oxidized lipids and auto fluorescent vitamin A derivatives, by secreting them as lipoprotein particles. However, in AMD, these particles are abnormally retained (23), exacerbating further lipid accumulation (15). Overall, dysregulated lipid metabolism and oxidation, play critical roles in RPE dysfunction and are key contributors to the pathogenesis of AMD.

### 2.2 AMD genetics provide an unbiased link to Factor H and complement

In 2005, several independent GWAS identified a significant association between AMD and the rs1061170 polymorphism in the gene encoding the complement regulator Factor H (*CFH*). This variation results in a Y402H substitution. Subsequent genetic studies uncovered additional variants in *CFH*, the C*FHR* gene cluster, the rs3750846 polymorphism in *ARMS2/HTRA1* locus, and 17 other genomic regions, all significantly associated with AMD (p < 5×10^-8^). The encoded proteins are involved in key biological processes such as complement activation and regulation, lipid metabolism, extracellular matrix remodeling, and angiogenesis (24–27).

Meta-analyses involving large cohorts of AMD patients and controls have expanded the list of AMD-associated genes, confirmed earlier findings, and provided deeper insights into the disease’s underlying pathology. Indeed, one study analyzing data from 16,144 AMD patients identified 34 genomic loci and 52 independent signals linked to AMD (16–18, 28–30). More recently, the International AMD Genomics Consortium conducted a comprehensive study involving 57,290 AMD cases and 364,430 controls. This analysis validated the 34 previously-identified loci, discovered 26 novel loci, and revealed substantial genetic variation across ethnic groups, particularly among individuals of African ancestry (31). Common single nucleotide polymorphisms (SNPs) have also been identified in other complement-related genes, including *CFI* (Factor I) (32), SNPs rs2230199 and rs1047286 in *C3* (33–35), and rs641153 in *CFB* (Factor B) and rs547154, rs9332739 in *C2* (36, 37). Recently, complement regulators such as CD46 and CD55 have also been implicated. The growing number of complement-associated genes underscores the critical role of dysregulated complement in AMD pathogenesis (24, 38). In addition, new risk variants have been identified in genes involved in lipid metabolism, such as rs429358 in *APOE* (apolipoprotein E) and rs2043085 in LIPC (hepatic lipase), further supporting the association of lipids with AMD (39).

To date, four major risk loci have been identified within *CFH*, each affecting different residues in the short consensus repeat (SCR) domains: rs800292 leading to V62***I*** in SCR1 (risk variant with red letter), rs1061170 leading to Y402***H*** in SCR7, rs2274700 with no exchange A473A in SCR8, and rs121913059 leading to R1210***C*** in SCR20. The Y402***H*** variant, located in the binding region of SCR7 and present in both Factor H and FHL-1, is particularly well-studied. Individuals homozygous for the Y allele (YY) exhibit normal risk, while heterozygous carriers (Y***H***) show moderate risk. Homozygosity for the H allele (***HH***) however increases the risk of developing AMD by approximately sevenfold. Further genetic studies have confirmed the association of the Y402***H*** polymorphism with AMD and identified additional variants in *CFH*, such as the intronic SNP rs1410996, which confers a level of risk (40–42). Risk alleles in one or multiple complement genes —particularly the Y402**H** variant in Factor H can increase an individual’s lifetime risk of AMD to up to 50% (43). In addition to genetic predisposition, environmental and lifestyle factors such as dyslipidemia and smoking further elevate the risk of developing AMD (44).

Further, a common chromosomal deletion encompassing the complement Factor H-related genes *FHR1* and *FHR3* has been shown to confer protection against AMD (25–27). Interestingly, this chromosomal deletion is also protective in IgA Nephropathy (IgAN) but conversely increases susceptibility to systemic lupus erythematosus (SLE) (45) and *Neisseria meningitidis* infection (46, 47).

Other Factor H-related proteins, such as FHR4 and FHR5, have also been implicated in AMD, with FHR4 being a lipid binding protein (48). The role of FHR4 remains controversial. One study found elevated levels of FHR4 in plasma of AMD patients (49), while another reported no association between FHR4 plasma levels and AMD progression or causality (50). Recent genetic evidence suggests a protective role for reduced FHR5 plasma levels in AMD. Studies (51, 52) identified rare, predicted loss-of-function variants in FHR5, including a missense variant associated with a reduced risk of AMD. Furthermore, systemic levels of FHR proteins — and the interplay of FHR1 with Factor H — appear to be relevant to disease pathology. Elevated plasma concentrations of FHR1, FHR2, and FHR4 have been observed in AMD patients, whereas Factor H levels remain unchanged (53).

The association between elevated risk of AMD and alterations in multiple complement genes strongly suggests a critical homeostatic role for the complement system in the eye. Dysregulation of this system can lead to local damage, chronic inflammation, and contribute to AMD pathogenesis.

## 3 Complement - a trigger of AMD pathogenesis and inducer of para-inflammation

**Figure 1:**
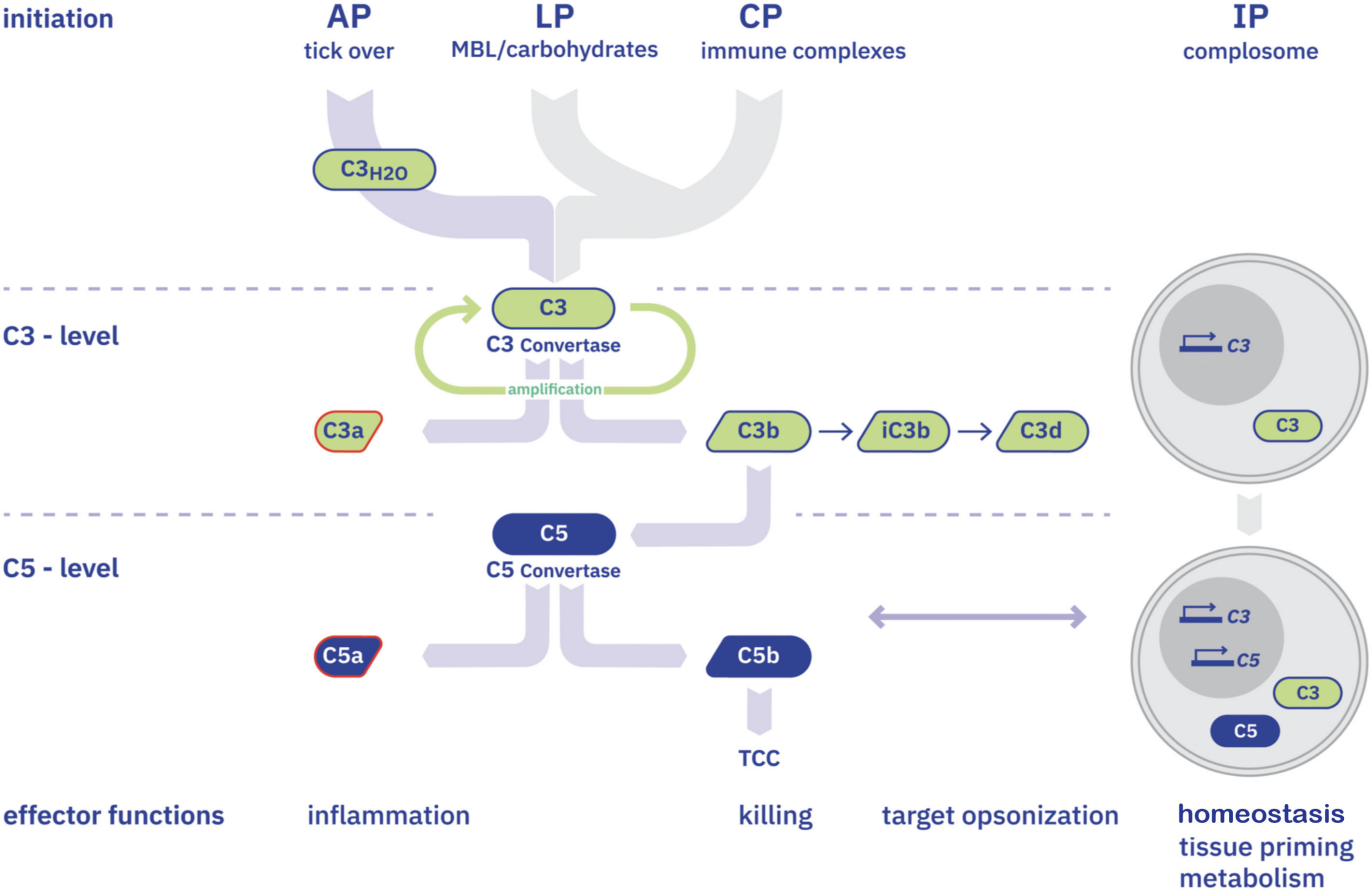
Complement activation pathways and effector functions The complement system can be activated both in the fluid phase and on biological surfaces through three primary pathways: the alternative pathway (AP), the lectin pathway (LP), and the classical pathway (CP). More recently, an intracellular complement pathway (IP) has also been identified, underscoring the role of complement in cellular homeostasis and metabolism. All activation routes converge at a central enzymatic hub—the C3 convertase. The formation of C3 and C5 convertases defines the two major effector stages of the complement cascade. Cleavage of C3 produces two key effectors: C3a, a potent anaphylatoxin with chemotactic and antimicrobial properties. C3b, which opsonizes target surfaces and is further processed into iC3b, C3dg, C3c, and C3d, facilitating immune recognition and phagocytic clearance. Cleavage of C5 generates C5a, a highly potent anaphylatoxin and chemoattractant, and C5b, which initiates the assembly of the membrane attack complex (MAC), also known as the terminal complement complex (TCC or C5b-9), leading to lysis of target cells. The intracellular pathway functions independently but corresponds with the extracellular cascade and is involved in processes such as tissue priming, cell survival, metabolic regulation, and the induction of adaptive immune responses.

The complement system is an evolutionarily conserved and essential component of innate immunity, playing a vital role in maintaining immune homeostasis and protecting the host from microbial infections. Upon activation, the complement cascade produces cytotoxic fragments and inflammatory mediators (**Figure 1**), which also participate in initiating and modulating adaptive immune responses.

Complement activation in the circulation and on surfaces occurs via three distinct pathways, each further supported by an intracellular complement. The alternative pathway is continuously and spontaneously active at low levels, providing immediate immune surveillance. The lectin pathway is triggered by the binding of mannose-binding lectins to carbohydrate structures on microbial surfaces. The classical pathway is initiated by the binding of complement component C1q to immune complexes, such as antigen–antibody aggregates.

All three pathways converge at the formation of the C3 convertases, which cleave C3 into C3a and C3b. If the cascade continues, C5 convertases are formed, leading to the cleavage of C5 into the potent anaphylatoxin C5a and C5b. C5b initiates the assembly of the terminal complement complex (TCC), also known as C5b-9, which forms pores in target cell membranes (54).

Complement activation can occur not only in the fluid phase in plasma, but in the vitreous fluid of the eye, as well as on altered self-surfaces—such as damaged or apoptotic cells—and on foreign surfaces, including those of invading bacteria and viruses. Under normal conditions, intact self-surfaces regulate complement and provide a local protective, non-inflammatory environment. These surfaces, which do not trigger complement activation, are safeguarded from the cytotoxic effects of the complement system.

### 3.1 The Complosome - intracellular complement and immune–metabolic regulation

The intracellular complement system, often referred to as the complosome, interacts with the extracellular complement cascade and functions as a critical immune–metabolic regulator within cells. It governs key processes such as cell survival, metabolism, and inflammatory responses —extending far beyond the classical extracellular roles of complement.

While the extracellular functions of complement have been extensively studied over decades (about 140 years), recent discoveries have revealed important physiological roles for intracellular complement in cellular homeostasis, metabolic regulation, tissue priming, and immune signaling (55, 56). Intracellular C3 activation was first identified in T cells, where C3 is cleaved in lysosomes by the protease cathepsin L, generating active C3a and C3b. C3a binds to C3a receptors on lysosomal membranes, triggering glycolysis and activating the mTOR pathway, both essential for T cell survival —a mechanism likely relevant across multiple cell types (56, 57).

Within the complosome, Factor H plays a pivotal role in regulating mitochondrial homeostasis, autophagy, metabolism, cell survival, and gene expression (57). In apoptotic cells, Factor H is actively internalized, and facilitating the generation of iC3b, exerting anti-inflammatory effects, and promoting the efficient clearance of apoptotic debris (58).

Similar to the extracellular cascade, intracellular C3 and C5 can be activated by specific proteases or by intracellularly assembled convertases (59–61). These pathways are tightly regulated and responsive to extracellular signals, contributing to cytokine production, immune signaling, and cellular equilibrium. Intracellular complement proteins can originate from cytoplasmic pools or be recruited from the circulation. Experimental evidence shows that plasma-derived Factor H as well as C3, C5, can traffic into intracellular compartments, suggesting a dynamic exchange between extracellular and intracellular complement systems (62–64). Importantly, intracellular C3a —which is essential for maintaining cellular homeostasis— cannot be functionally replaced by extracellular C3 or C3a, underscoring the unique and non-redundant role of the complosome in cell physiology (63).

### 3.2 Complement regulators Factor H and FHL-1

A network of plasma and membrane-bound proteins tightly regulate complement activation and the generation of effector molecules. These complement regulators act in both the fluid phase and on cell surfaces to ensure that activation is spatially and temporally restricted, thereby preserving the integrity of healthy self-tissue. Intact self-surfaces are protected by these regulators and do not trigger complement activation. In contrast, necrotic cells and foreign surfaces typically lack such regulators and protection, allowing complement activation to proceed, resulting in C3b deposition, opsonization, and ultimately elimination via phagocytosis. Persistent or uncontrolled complement activation can lead to tissue damage and chronic inflammation. Among the key regulators of the alternative pathway are Factor H and FHL-1, both of which function in the fluid phase and can bind to biological surfaces.

#### 3.2.1 Structure and function of Factor H

Factor H and FHL-1 are encoded by *the Factor H gene* located on chromosome 1q32. They are central regulators of the alternative pathway, controlling initiation, and particularly the amplification loop (**Figure 1**). Their regulatory functions include decay acceleration, which means displacement of the Bb fragment from the C3 convertase (C3bBb) and thereby destabilizing the convertase complex, and cofactor activity leading to the cleavage of C3b into inactive iC3b by the serine protease Factor I (65, 66). Additionally, Factor H and FHL-1 regulate the low-level, spontaneous “tick-over” activation of C3 in plasma, maintaining C3 homeostasis. In the absence of Factor H and FHL-1, uncontrolled C3 activation leads to complete conversion to C3b in the fluid phase, causing pathology.

**Figure 2:**
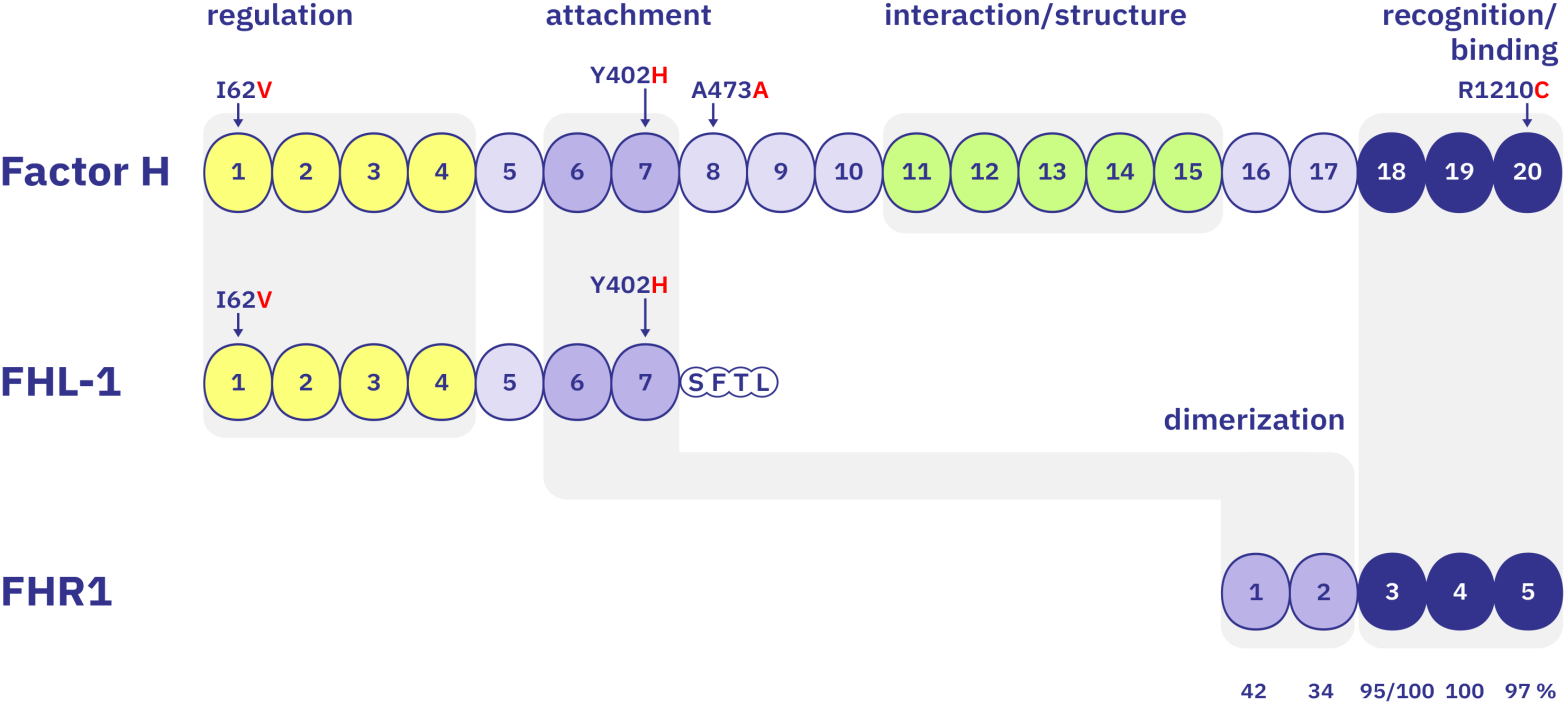
Domain structure of Factor H and homology with FHL-1 and FHR1 All molecules are composed of repetitive short consensus repeat (SCR) domains. Factor H consists of 20 SCR domains, organized into four functional segments: Segment A (SCR 1–4, yellow) represents the N-terminal regulatory region which binds to C3 and C3b, mediates coFactor activity and decay acceleration and includes the AMD-associated I62***V*** polymorphism (risk variant shown in red). Segment B (SCR 6–7, light blue) includes the surface attachment region, binds to heparin, GAGs, sialic acids, and MDA and contains the central AMD-associated Y402***H*** polymorphism. Segment C (SCR 11–15, green) mediates low-affinity binding to C3, heparin, and GAGs and likely contributes to structural flexibility and conformation. Segment D (SCR 18–20, dark blue) includes the C-terminal recognition region, binds to C3b, C3d, and surface ligands such as heparin, GAGs, and MDA and includes AMD-associated polymorphism R1210C (SCR20). FHL-1 consists of the first seven SCR domains of Factor H with a unique C-terminal extension of four amino acids (SFTL). This protein shares Segment A (SCR 1–4) and Segment B (SCR 6–7) with Factor H, functions as a complement regulator and is expressed by RPE cells. FHR1 contains five SCR domains. SCR 1–2 contain a dimerization motif and share moderate sequence homology with Factor H SCR 6–7 (42% and 34%, respectively). SCR3 exists in two allelic variants differing by HLE or YVQ motifs and shares 100%/95% identity with SCR18 of Factor H, respectively. SCR4 is identical to SCR19 of Factor H. SCR5 shares 97% homology with SCR20 of Factor H, differing by two residues (LA in FHR1 vs. SV in Factor H).

Factor H is primarily synthesized in the liver, but is also expressed locally, for example by RPE cells in the eye (67). It is a 150 kDa glycoprotein composed of 20 short consensus repeats (SCR), also known as complement control protein modules. The protein adopts a flexible, extended conformation and interacts with multiple ligands, including C3b, glycosaminoglycan (GAGs) like heparin, and malondialdehyde (MDA) (54, 68– 69, 70) (**Figure 2**).

Factor H contains four distinct regions: Segment A (SCR1–4) binds to C3b, particularly when it is surface-bound, and mediates regulatory activity. Segment B (SCR6–7) represents a surface recognition domain that binds to heparin, GAGs, sialic acids, and MDA but does not bind C3b. Segment C (SCR11–15) provides low-affinity interactions with C3b and GAGs and likely contributes to structural flexibility. Segment D (SCR18–20) harbors a key recognition domain that binds to C3b, C3d, and surface ligands such as heparin, GAGs, and MDA on modified self-surfaces.

The four functional segments of Factor H vary in length, comprising between two and five SCR domains, and bind a diverse array of ligands —including the activation product C3b and degradation products iC3b and C3d, surface constituents, and oxidized lipids. Segment C also contains a structural element that contributes to the protein’s conformational flexibility. The two N-terminal segments (A and B) form single-point interactions with either C3, C3b, or surface ligands, respectively. In contrast, the middle segment (C) and the C-terminal segment (D) possess overlapping binding sites for both, C3 variants and surface ligands, enabling multivalent interactions and enhanced regulatory capacity (68, 71).

**Figure 3:**
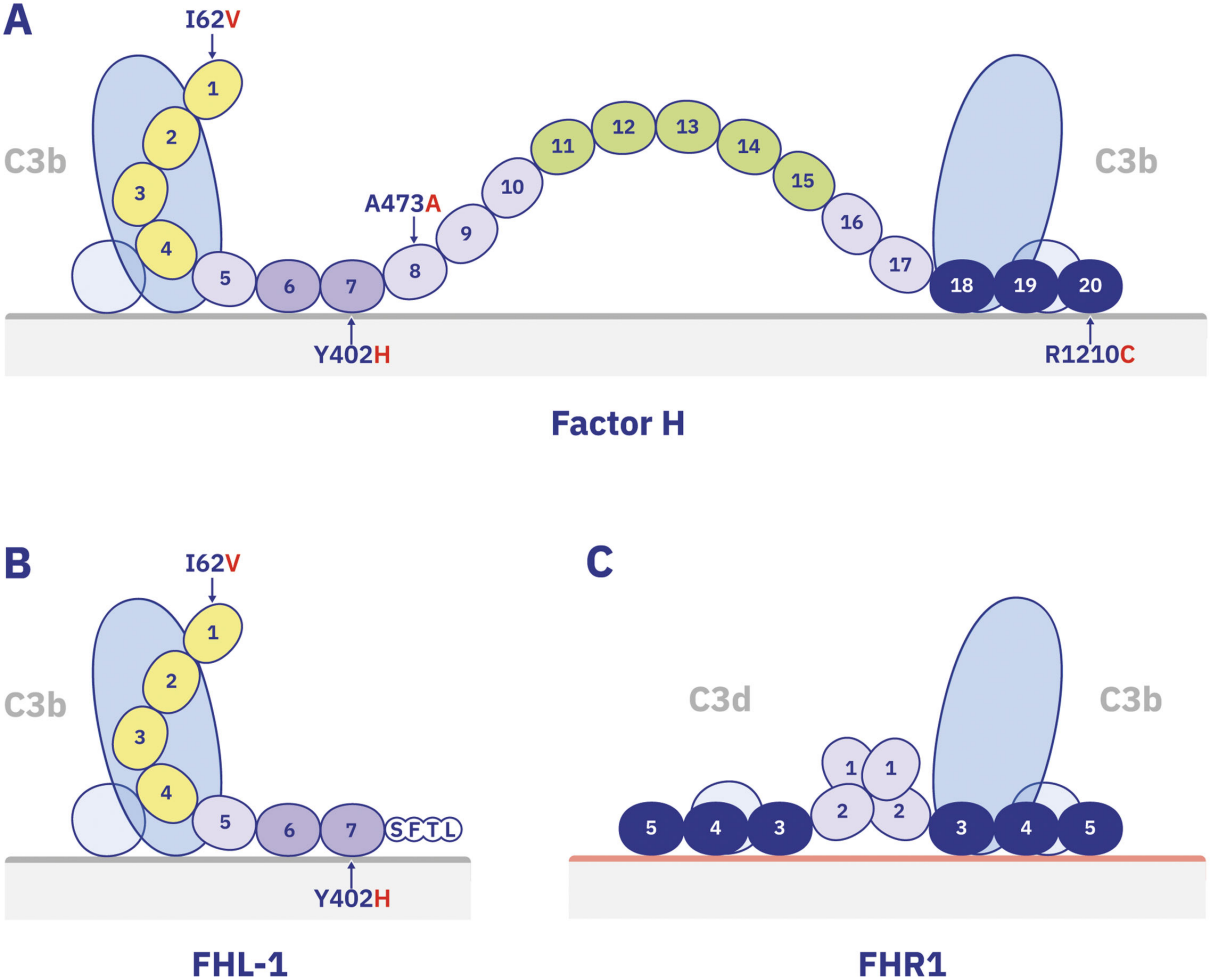
Model of surface interactions of Factor H, FHL-1, and FHR1 with C3 fragments on host surfaces **(A)** This panel illustrates a hypothetical model of Factor H binding to surface-deposited C3b in its extended, flexible conformation. The N-terminal regulatory region (Segment A, yellow) binds to C3b, while the first surface attachment region (Segment B, light blue) engages host surface markers. Simultaneously, the C-terminal recognition region (Segment D, dark blue) may form a third contact with surface ligands such as GAGs or MDA. Segment C (green) may adopt a hairpin-like loop structure. Via its C-terminal domain Factor H also binds C3d. Note: C3b is depicted with two color-coded regions—representing the binding sites for the N-terminal and C-terminal domains of Factor H **(B)** FHL-1, the splice variant of the *CFH* gene, shares the same binding domains for C3b (SCR1–4) and surface ligands (SCR6–7). Due to these shared sites, FHL-1 may compete with full-length Factor H for binding to C3b and host surfaces. However, its shorter length and single attachment domain result in more restricted surface interaction. **(C)** FHR1 binding to C3b and C3d. FHR1, a dimeric protein, shares a nearly identical C-terminal domain with Factor H It binds to both C3b and C3d. However, due to differences in surface-binding domains and structural configuration, FHR1 and Factor H likely bind to slightly distinct surface environments, influencing their functional roles in complement regulation.

The size of Factor H, combined with its high structural flexibility, allows Factor H to engage with a variety of surface topologies and select specific binding sites. Its conformation may determine which contact segments are exposed and accessible. Depending on which surface-binding segment is engaged, the N-terminal regulatory segment A can be positioned at varying distances from the surface. This spatial variability could enable layer-specific complement regulation (**Figure 3A**). Genetic data strongly suggest that the attachment segment (Segment B) and the recognition segment (Segment D) are relevant to AMD pathology. Binding of both segments might be influenced either by FHL-1 (**Figure 3B**) or by FHR1 (**Figure 3C**), which share either the attachment segment (FHL-1) or the recognition segment (FHR1), respectively.

#### 3.2.2 FHL-1, a functional splice variant of *the Factor H gene*

Factor H-like protein 1 (FHL-1) is encoded by *CFH* and the encoded protein consists of the first seven SCR domains of Factor H which are followed by a unique C-terminal amino acids tail - SFTL. This 42 kDa protein retains the regulatory segment A (SCR1-4) and the attachment segment B (SCR6-7), enabling C3 convertase activity regulation as well as displaying decay acceleration and binding to surfaces via SCR6-7.

FHL-1 is an efficient complement regulator in its own right and plays a particularly important role in tissues, such as the retina, where it may be more prevalent than full-length Factor H. Initially identified as fluid-phase complement regulators, both Factor H and FHL-1 are now known to bind to biological surfaces, including altered tissue membranes and apoptotic as well as necrotic cells. FHL-1, being shorter, combines the C3 regulatory segment with a single surface attachment domain (SCR7). Thus, FHL-1 shows more restricted surface interaction than Factor H, which has three separate surface sites. Nevertheless, both proteins are capable of binding to modified self-surfaces, such as retinal RPE cells and the RPE/retinal interface, where they inhibit complement activation at the C3 convertase level (70, 72).

#### 3.2.3 Non-canonical functions of Factor H and FHL-1

**Figure 4:**
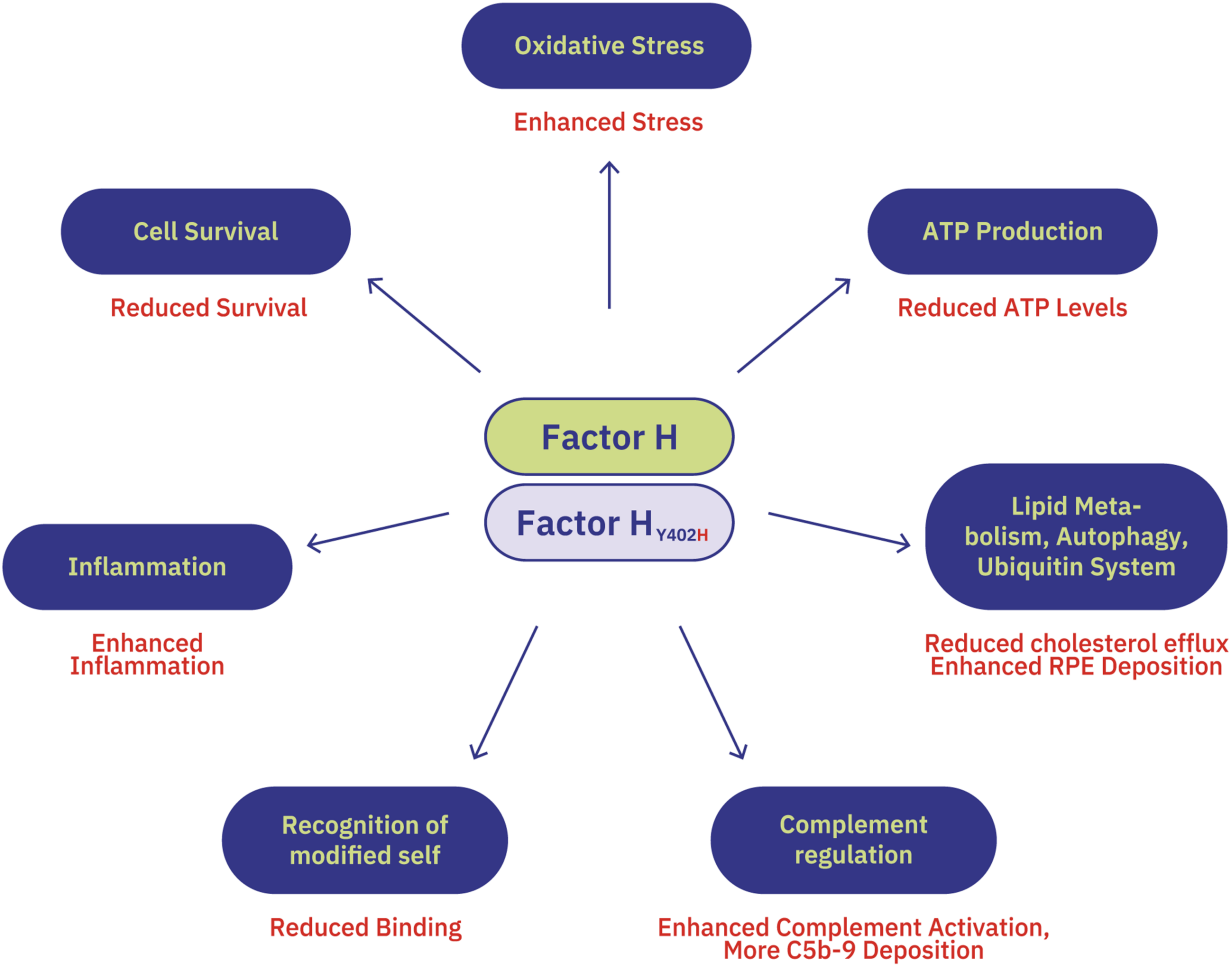
Multifunctional roles of Factor H and impact of the 402H risk variant Factor H serves as the central regulator of complement activation, particularly at the level of the C3 convertase, and also mediates a range of non-canonical cellular functions. These include regulation of inflammation, cell survival, oxidative stress, ATP production, and lipid metabolism. The 402***H*** risk variant of Factor H contributes to disease susceptibility by either enhancing or impairing these functions, particularly in the context of AMD. These expanded roles of Factor H —beyond complement control— highlight its importance in maintaining cellular homeostasis, especially in tissues such as the retina. Many of these functions have been elucidated through studies using iPSC-derived RPE cells from AMD patients, recombinant Factor H, and gene silencing approaches.

While fFctor H and FHL-1 are well-established regulators of the complement system—particularly at the level of the C3 convertase—emerging evidence reveals that these proteins also perform non-canonical functions beyond complement regulation (**Figure 4**). These roles are especially relevant in the context of intracellular Factor H, which has been implicated in a range of cellular processes.

Recent studies have identified novel functions for Factor H and likely FHL-1 in: inflammation modulation, cell survival, oxidative stress enhancement, ATP production regulation, and lipid metabolism. Many of these non-canonical activities were uncovered through investigations in the AMD context, involving induced pluripotent stem cell-derived retinal pigment epithelial cells (iPSC-RPE) from AMD patients (see chapter 3.2.5. for more details). Functional insights were gained by analyzing the effects of recombinant Factor H, AMD-associated risk variants, and gene silencing approaches targeting *Factor H expression*. Importantly, these findings show that Factor H and FHL-1 are not only extracellular regulators but also play intracellular roles in maintaining cellular homeostasis, particularly in tissues vulnerable to oxidative damage and metabolic stress, such as the retina.

Relevant for AMD is that Factor H, as well as FHR1 can modulate monocyte/macrophage activation and affect downstream cytokine release, beside complement activation. Both, Factor H and FHR1 interact with lipid-containing structures such as oxidized lipoproteins (70, 73–75) and extracellular matrix components (54, 68, 69), which is particularly relevant in chronic inflammatory diseases such as AMD. However, recent data suggest that while Factor H dampens the immune response on self surfaces (70), FHR1 is an activator of the inflammatory immune response (74, 75). Through these different functional activities Factor H and FHR1 influence the local inflammatory microenvironment, induce immune cell recruitment, and substantially contribute to the regulation of tissue homeostasis and inflammation in response to cellular damage.

#### 3.2.4 Expression of Factor H and complement components in the eye

In the retina, Factor H and FHL-1 are synthesized by RPE cells and bipolar cells, with expression levels increasing in response to drusen-mediated complement activation. Immunohistochemically analyses have shown that Factor H is expressed in RPE cells, choroidal endothelial cells surrounding the choriocapillaris, the ganglion cell layer, and the plexiform layers (at lower levels), and is strongest in the fovea compared to the macula and peripheral retina (76).

These studies, which primarily focused on Factor H, did not distinguish between Factor H and FHL-1 and did not include tools specific for FHL-1. In addition to Factor H and FHL-1, RPE cells also secrete C3, Factor B, and Factor I, but notably not FHR1 (77). This supports the role of RPE cells as a local source of complement proteins, thereby contributing to immune surveillance and retinal homeostasis.

Expression of Factor H in ocular tissues has been confirmed through **s**ingle-cell RNA sequencing, *in vitro* protein expression analyses, and immunohistochemistry. In aqueous humor from patients with neovascular AMD, Factor H levels were only moderately elevated compared to healthy controls (0.16 vs. 0.13 µg/mL). Similarly, C3 (0.78 vs. 0.72 µg/mL) and Factor I (0.17 vs. 0.11 µg/mL) levels remained comparable. However, activation markers C3a and Ba were significantly increased (by at least 50%) in AMD specimens (C3a: 21.0 → 33.0 ng/mL; Ba: 11.5 → 22.8 ng/mL) (78).

Importantly, the concentrations of Factor H, C3, and Factor I in the aqueous humor are substantially lower than in plasma, suggesting limited diffusion from the choroidal circulation and reinforcing the concept of the intact retina as an immunologically privileged site with local complement production.

#### 3.2.5 Impact of Factor H risk variants

After the identification of high-risk variants in the *Factor H* gene associated with the development of AMD, research has increasingly focused on elucidating the functional consequences of these modifications. Collectively, the risk variants alter both the extracellular and intracellular roles of Factor H, extending its pathological impact well beyond complement regulation on cell and tissue surfaces.

Factor H and its alternatively spliced form FHL-1 share two major AMD-associated risk loci: the I62***V*** variation located in SCR1 (*CFH*SNP rs800292) and the Y402***H*** variation in SCR7 (*CFH* SNP rs1061170) (16, 28). Structural analysis of Factor H SCR1–4 in complex with C3b revealed that SCR1 is positioned largely outside the Factor H–C3b interface (79). As indicated in **Figure 3**, the I62V variation in SCR1 is therefore unlikely to directly influence C3 or C3b binding. However, this substitution —potentially modulated by tRNA abundance —may affect protein translation efficiency, thereby altering *Factor H* gene expression levels both in the circulation and within the cytoplasm (80–84).

The second major AMD-associated polymorphism, Y402H, located in the attachment region (segment B), is shared by both Factor H and FHL-1. Both risk variants—Factor H_402H_ and FHL-1_402H_— exhibit reduced binding affinity to necrotic or damaged cellular surfaces, to heparin, to glycosaminoglycan (GAG), and to oxidized lipids and membranes. Importantly, these variants do not significantly alter C3 binding. Consequently, the decreased affinity of Factor H and FHL-1 for oxidized surfaces in the retina results in impaired local complement regulation (70, 73, 85).

Interestingly, population-specific associations have been observed. In Caucasian cohorts, the Y402***H*** variant shows a strong correlation with AMD susceptibility, whereas in Asian populations, the I62V variant is more strongly associated with the disease. This observation indicates that genetic background and population-specific allele frequencies contribute to AMD risk. Beyond their canonical role in complement control, Factor H and FHL-1also exert non-canonical functions that are influenced by the Y402***H*** polymorphism. Both risk variants modulate key cellular processes, including inflammatory signaling, cell survival, binding to specific sites of damaged RPE cells and responses to oxidative stress (70, 73, 86–90).

In induced pluripotent stem cell-derived retinal pigment epithelial (iPSC-RPE) cells, either the expression of AMD risk variants or the silencing of endogenous Factor H increases susceptibility to oxidative stress and reduces cellular viability (84) (**Figure 4**). Exogenous supplementation of Factor H protects RPE cells from lipid oxidation product-induced damage (87), whereas Factor H-silenced iPSC-RPE cells fail to respond to such protective effects (67). In both Factor H-deficient and Y402***H***-expressing iPSC-RPE cells, mitochondrial function and energy metabolism are markedly impaired, with reduced ATP production and glycolytic activity (91, 92). Furthermore, lipid metabolism is disrupted (86, 93–97), leading to intracellular lipid accumulation and metabolic dysregulation (**Figure 4**). The Factor H_402H_ variant promotes lipid accumulation in retinal pigment epithelial (RPE) cells. Under physiological conditions, Factor H limits the uptake of oxidized low-density lipoproteins (oxLDLs) in ARPE-19 cells (98). However, the Y402H variant disrupts this protective function due to its reduced binding affinity for oxidized surfaces, including oxLDLs and malondialdehyde (MDA)-modified proteins (70, 73, 85).

Exposure to oxidized photoreceptor outer segments further downregulates Factor H expression in RPE cells, increasing the risk of oxidative product accumulation and excessive complement activation (99). In addition, large high-density lipoprotein (HDL) particles isolated from AMD patients have been found to contain Factor H, suggesting that Factor H may associate with lipoproteins to suppress inflammation induced by oxLDLs (100).

The two Factor H variations A473A (rs2274700) in SCR8 and R1210C in SCR20 may affect Factor H function without changing FHL-1. It has been suggested that the synonymous A473A substitution in SCR8 modulates *CFH* expression and thereby alters Factor H protein levels (101, 102). In contrast, the R1210***C*** variant located within segment D has a pronounced effect on surface recognition. This became evident when the same substitution was identified as a rare variant causing atypical hemolytic uremic syndrome (aHUS) (103–105). R1210C is an unusual mutation as it promotes the formation of covalent complexes between Factor H and human serum albumin, mimicking partial Factor H deficiency (104). The recognition segment D of Factor H mediates interactions with both C3b/C3d and host cell surface components and therefore critical for Factor H*-*mediated complement regulation on self-surfaces. Reduced availability of Factor H binding resulting from the R1210C substitution compromises complement control on host tissues and thereby increases susceptibility to AMD.

Recent findings have further expanded the understanding of Factor H biology, revealing that Factor H is also active within the cytoplasm, where it negatively regulates intracellular C3 activation and prevents excessive complement-mediated priming of cells (106). Similarly, Mahajan et al. (107) demonstrated that intracellular Factor H inhibits the nuclear translocation of NF-κB in kidney endothelial cells. As NF-κB orchestrates numerous pro-inflammatory pathways, its regulation by Factor H is crucial for maintaining cellular homeostasis and controlling inflammation.

Together, these findings indicate that the Y402H variant impairs the ability of RPE cells to clear oxidized lipids, promoting their intracellular accumulation. This lipid build-up, combined with local complement dysregulation, contributes to cellular stress, chronic inflammation, and drusen formation—hallmarks of AMD pathogenesis. By linking altered lipid handling to complement over activation, these data provide a mechanistic explanation for how the Y402***H*** variant increases susceptibility to retinal degeneration.

Taken together, the biological consequences of the Y402H and other Factor H variations are broad, influencing both extracellular and intracellular complement regulation by Factor H and FHL-1. Impaired complement control promotes excessive activation, while loss of ligand-binding and immunomodulatory properties exacerbates oxidative stress, debris accumulation, and chronic inflammation. This dual impairment of Factor H function in AMD provides a plausible mechanistic explanation for the strong disease association of these variants. Furthermore, these insights suggest that therapeutic complement inhibition alone may not fully restore the broader immunomodulatory and cytoprotective roles of Factor H. This is underlined by the fact that Factor H also negatively controls choroidal neovascularization as efficiently as currently used anti VEGF treatment, as shown in a murine model of CNV (108).

### 3.3 FHR1, the inflammatory modulator

The chromosomal deletion of the gene encoding FHR1 is protective in AMD. The FHR1 encoding gene resides within the regulators of complement activation cluster on human chromosome 1q32, and its encoded protein shares structural homology with Factor H and FHL-1 (109) (**Figure 2**). FHR1 consists of five SCR domains and forms homodimers via its N-terminal SCR1–2 domains (**Figure 3C**). At elevated concentrations, FHR1 can inhibit C5 convertase activity, both *in vitro* and *in vivo* (110, 111). However, unlike Factor H and FHL-1, FHR1 lacks cofactor and decay-accelerating activity, and therefore does not regulate C3 convertase.

The C-terminal recognition domains of FHR1 and Factor H exhibit high sequence homology, differing by only two amino acids in their most distal SCR- SCR5 of FHR1 and SCR20 of Factor H. Both proteins bind to C3b, C3d, heparin and necrotic surfaces, but with distinct binding affinities. Notably, FHR1 binds C3d with higher affinity than C3b and does not bind glycosaminoglycans on healthy self-surfaces (112). FHR1 preferentially binds to complement-activated surfaces and shows strong affinity for oxidized LDL (oxLDL) (70, 73, 74). These differences in binding profiles suggest distinct roles and timing for FHR1 and Factor H. While Factor H targets modified self-surfaces with C3b deposition, regulating C3 convertase activity and C3b processing, FHR1 binds to surfaces enriched in C3d, promoting macrophage-mediated inflammation—indicating a sequential and complementary action. Surface-bound FHR1 interacts with monocytes via the EMR2 receptor, triggering inflammasome activation and the release of pro-inflammatory cytokines such as IL-1β and TNF-α (74). Recent studies support this model, showing that FHR1 bound to necrotic RPE cells enhances monocyte infiltration and activation in AMD animal models (77). This para-inflammatory response may explain why genetic FHR1 deficiency is protective not only in AMD but also in atherosclerosis (26, 75). In the damaged retina, FHR1, derived from the choroid may compete with Factor H for binding sites, potentially hindering Factor H’s regulatory function. Whether FHR1 directly interferes with Factor H binding or modulates it indirectly remains under investigation. The hypothesis that FHR1 deletion enhances Factor H function is compelling but has not been confirmed *in vivo*.

## 4 AMD pathophysiology – a model for sequential events

Understanding the underlying pathogenesis of AMD remains an ongoing challenge, as the disease is multifactorial and complex. One prevailing hypothesis suggests that hemostatic balance in the aging eye is disrupted due to reduced clearance of metabolic waste. Choroidal thinning is debated as a risk factor (113–115). This impairment is exacerbated by an increased lipid burden in the retina and the accumulation of extracellular deposits known as drusen.

These conditions likely hinder the bidirectional transport of oxygen, nutrients, and waste products between the RPE and the choroid. Once deposited, lipids undergo oxidation, triggering complement activation and initiating inflammatory processes. These are further amplified by bystander cells. At this stage, complement regulation—particularly by Factor H and FHL-1—is crucial to maintain homeostasis and limit tissue damage.

**Figure 5:**
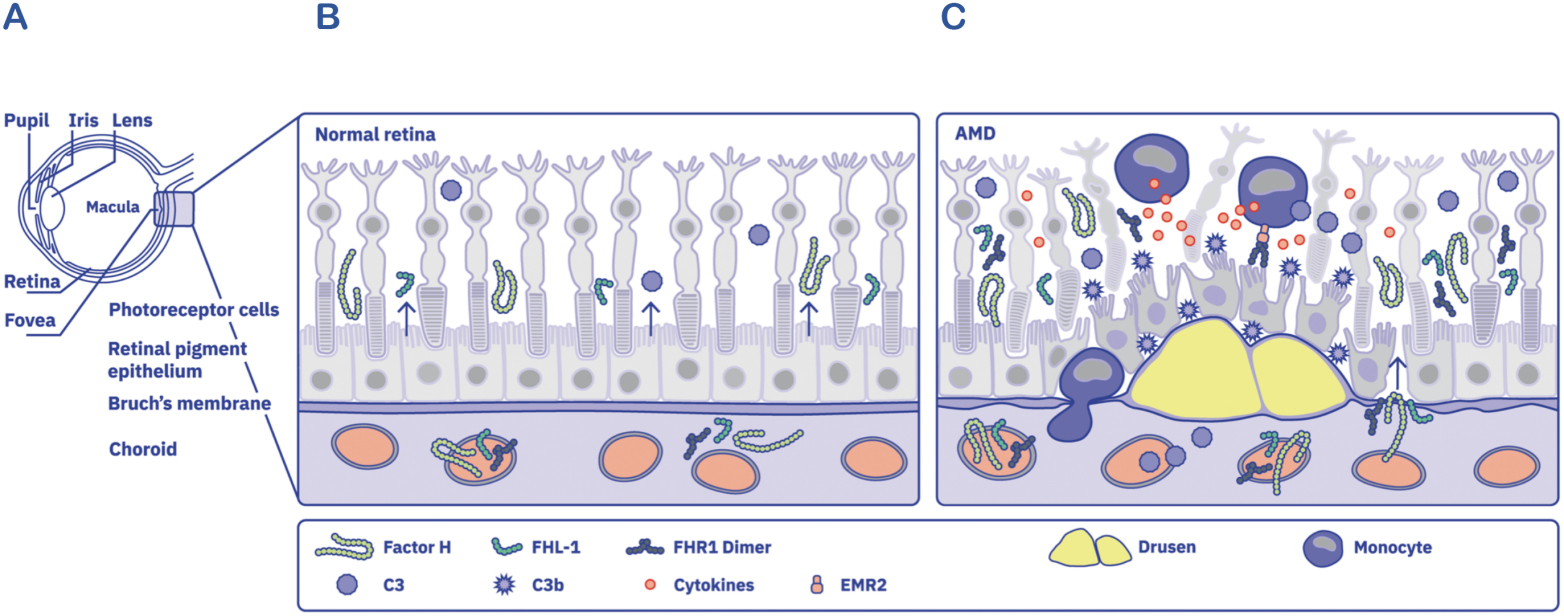
Composition of the eye with the healthy and AMD conditions, highlighting complement targets and integrating Factor H, FHL-1, and FHR1 **(A)** Anatomical structure of the human eye (Left panel) **(B)** Representation of a healthy retina, showing the intact retinal pigment epithelium (RPE) layer and photoreceptors (middle panel). The Bruch’s membrane separates the RPE from the underlying choroid. RPE cells secrete complement components including Factor H, FHL-1, and C3, but not FHR1. In contrast, Factor H, FHL-1, C3, and FHR1 are present in the plasma within the choroid. **(C)** Pathological scenario in AMD, characterized by damaged RPE cells and photoreceptors, and disruption of the RPE layer (right panel). Drusen deposits from beneath the RPE within the compromised Bruch’s membrane. Activated innate immune cells, such as monocytes, migrate from the choroid into the retinal space. Concurrently, plasma proteins —including Factor H, FHL-1, FHR1 dimers, and C3—leak from the choroid. FHR1 dimers bind to damaged RPE cells and activate infiltrating monocytes via EMR2 receptors, amplifying the inflammatory response. Activated monocytes release cytokines and chemokines, intensifying para-inflammatory responses in the retina.

However, in AMD, risk variants of Factor H and FHL-1 impair both extracellular and intracellular functions, and non-canonical roles reducing the efficiency of complement control through multiple mechanisms. Over time, this dysregulation leads to a cascade of events, including lipid accumulation, oxidative stress, sustained complement activation, chronic inflammation and ultimately, disruption of the retinal barrier. This breakdown results in the leakage of fluids and proteins from both the retina and the choroid, culminating in retinal atrophy. This degenerative state promotes the massive infiltration of proteins and monocytes into the retinal space. Once FHR1 enters this space from the choroid, via a damaged Bruch’s membrane inflammation is further intensified, and infiltrating blood-derived monocytes become activated (**Figure 5**).

## 5 AMD therapy

In addition to conservative management strategies for AMD such as reducing modifiable risk factors like smoking (116), excessive sunlight exposure, and obesity. Antioxidant vitamins, like proposed in the AREDS 2 study (44), could have positive effects on subgroups of AMD patients (117). Adherence to a Mediterranean diet and engaging in regular physical activity can have positive impacts on AMD progression (117, 118). Given that complement system dysregulation plays a key role in AMD pathogenesis, current therapeutic approaches include complement inhibitors that target various levels of the cascade (**Table 1**). Emerging strategies are also investigating the potential of endogenous natural regulators to restore complement homeostasis, as well as dual-acting compounds that combine inhibitory and regulatory functions. Furthermore, there is growing interest in multi-target therapies, including dual or triple-action inhibitors and combination treatments. To date, two complement inhibitors have been approved for the treatment of GA in AMD, each targeting distinct levels of the complement cascade.

**Table 1:** Landscape of complement inhibitors for therapy of AMD.

Compound	Company	Type	Level	Target	Effect	Application	Clinical trial #	Status AMD	Comment
pegcetacoplan	Apellis	peptide		C3	all pathways	intra vit	NCT03525600 NCT03525613 NCT04770545	approved	approved PNH & C3G
iptacopan	Novartis	small molecule		Factor B	AP & amplification loop	oral	NCT05230537	phase II	approved PNH, primary IgAN, & C3G
CPV-104	Eleva	recombinant protein		Factor H	AP & amplification loop	intra vit	EUCT2024 517992-19-00	phase I	
VOY-101	Perceive Bi-oherapeutics	AAV vector coding Factor H		Factor H	AP & amplification loop	intra vit	NCT05979714	phase I	
4D-175	Aevitas /4DMT	AAV vector coding short Factor H	C3	Factor H	AP & amplification loop	intra vit		preclinical	
ANX007	Annexon	mAB		C1q	CP	intra vit	NCT04656561	phase II	
GT005	Novartis	AAV vector		Factor I	AP/CP	sub retinal	NCT04437368	stopped	
B-LRx	Ionisis/Roche	siRNA		Factor B	AP	subcutan	NCT03815825	stopped	
CLG561	Alcon (Novartis)	mAB		Properdin	AP	intra vit	NCT01835015 NCT02515942	stopped	
danicopan	Alexion	small molecule		Factor D	AP & amplification loop	oral	NCT05019521	stopped AMD	approved PNH
lampalizumab	Roche	mAB		Factor D	AP & amplification loop	intra vit	NCT02247479	stopped	
avacin-captad Pegol	Zimura	aptamer	C5	C5	terminal pathway	intra vit	NCT02686658 NCT04435366	approved	
LFG316	Novartis	mAB		C5	terminal pathway	iv	NCT01527500	stopped	
eculizumab	Alexion	mAB		C5	terminal pathway	intra vit	NCT00935883	stopped	
AAVCAGsCD59	Hemera Bioscience / Janson	AAV vector	TCC	CD59	terminal pathway	sub retinal	NCT03585556	stopped	

### 5.1 Complement inhibition in GA: current therapies and emerging strategies

The C3 inhibitor pegcetacoplan (NCT04770545), and the C5 inhibitor avacincaptad Pegol (NCT04435366) have been approved by the FDA for the treatment of GA, both are administered via intravitreal injection (119, 120). These agents have demonstrated efficacy in reducing the progression of atrophic lesions in the retina.

In particular, pegcetacoplan-treated eyes with GA lesion margins ≥250 μm from the foveal center showed a directionally slower decline in visual acuity (121, 122). These findings reinforce the therapeutic potential of complement inhibition in GA and support further exploration of inhibitors targeting for other components of the complement cascade, as well as regulators capable of rebalancing complement activity. There is also growing interest in alternative molecular formats and delivery routes. This positive clinical impact has intensified research into pharmacological agents that selectively target complement activation at specific levels and anatomical sites.

Therapeutic success at the level of C3, exemplified by pegcetacoplan, further confirms the central role of complement activation in AMD pathogenesis. Inhibition of complement-mediated inflammation and C3b opsonization is essential for disease control.

### 5.2 Factor H-based therapies

Given the central role of Factor H in regulating upstream complement activation and maintaining retinal cell homeostasis, there is significant interest in leveraging this endogenous regulator for therapeutic purposes. Early clinical and preclinical studies have explored the use of Factor H to modulate complement activity in the eye. These approaches include delivery of full-length or truncated Factor H proteins directly into the retina, as well as gene therapy strategies to induce Factor H expression in retinal cells. Additionally, dual-acting combination therapeutics are under development—such as engineered proteins that combine Factor H’s regulatory function with C5-blocking or C3d-targeting monoclonal antibodies.

Factor H (CPV-104) is developed by the company Eleva and represents a recombinant, full-length human Factor H protein produced in moss and optimized for therapeutic use. Phase I trial (EUCT2024-517992-19-00) is ongoing. When administered via intravitreal injection in animal experiments, CPV-104 retains the full regulatory capabilities of plasma Factor H, effectively inhibiting overactive complement and dampening local inflammation in the retina (123).

In a mouse model of light-induced retinal damage, CPV-104 significantly attenuated retinal degeneration and modulated the retinal immune response. It counteracted gliosis and reduced inflammation, highlighting its potential as a novel therapeutic strategy for AMD (123).

### 5.3 Gene therapy approaches targeting Factor H

Voy-101 developed by Perceive Biotherapeutics employs a gene therapy strategy to induce long-term expression of full-length Factor H in retinal cells. Delivered via intravitreal injection, the gene vector is designed to enable sustained local production of Factor H, aiming to restore complement regulation in the retina. The compound is tested in clinical trial phase I (NCT060087458). 4D-175 is a codon-optimized, truncated version of Factor H delivered using a retinotropic adeno-associated virus (AAV) vector (R100), developed by 4DMT and Aevitas. Upon intravitreal injection, the engineered protein includes: Segment A: the regulatory domain, Segment B: the attachment domain, and Segment D: the recognition domain. This therapy is being developed specifically for GA secondary to dry AMD and preclinically tested.

.

### 5.4 Fusion proteins combining regulatory Factor H segments with monoclonal antibodies

Two innovative approaches aim to combine Factor H’s regulatory activity with monoclonal antibodies (mAbs) targeting the terminal pathway or directing the regulatory activity to local sites of complement activation. KP104 combines the regulatory segment of Factor H (SCR1–5) with a C5-binding and TCC-blocking mAb as described in (124). This dual-action biologic is designed to regulate the proximal alternative pathway and simultaneously inhibit the terminal pathway. Developed by Kira Pharma, KP104 is currently being evaluated for renal diseases and is not yet applied to AMD. The compound is administered via intravenous or subcutaneous injection. A related bispecific inhibitor developed by Q32 Bio (www.Q32bio.com), ADX-097 fuses the same regulatory segment of Factor H with a humanized mAb targeting C3d. This design enables targeted complement regulation at tissue sites with marked by C3b/C3d deposition (125). ADX-097 is also being investigated for renal diseases and has not yet been applied to AMD. The program is now continued by Akebia Therapeutics.

### 5.5 Other complement inhibitors in clinical development for AMD

ANX007 developed by Annexon Inc. is a humanized monoclonal antibody that binds to the globular heads of C1q, thereby blocking the initiation of the classical complement pathway (126). Administered via intravitreal injection, ANX007 has shown promise in preclinical monkey studies and is currently in Phase II trials (NCT04656561) for GA. Ionis-Factor B-LRx developed by Ionis Pharmaceuticals/Roche is a liver-targeted antisense oligonucleotide (siRNA) that reduces the expression of Factor B, thereby inhibiting systemic alternative pathway activation. It is administered subcutaneously and was tested in Phase II trials for dry AMD (NCT03815825), however the compound failed to reach the endpoints. Iptacopan (Novartis) is an orally administered small molecule that inhibits Factor B, blocking both initiation and amplification of the alternative pathway. Already approved for C3G (EMA, May 2024; FDA, March 2025), primary IgA Nephropathy (2024), and Paroxysmal Nocturnal Hemoglobinuria (2023), iptacopan is now being evaluated in a Phase II trial (NCT05230537) for early and intermediate AMD.

### 5.6 Complement gene therapy targeting other regulators

GT005 (PPY988), developed by Gyroscope Therapeutics (now part of Novartis), is an AAV-based gene therapy designed to express Factor I in the retina following sub retinal injection. In a Phase II clinical trial, GT005 led to a twofold increase in vitreous Factor I levels and a marked reduction in Ba and Bb levels, with minimal changes in C3a and C3b/iC3b. These findings suggest that Factor I expression modulates early alternative pathway activation, particularly at the level of tick-over. Proteomic analyses confirmed the expression of multiple complement proteins, including Factor I and Factor H, in the eye (127). However, development of GT005 as a treatment of AMD was stopped after phase II clinical trial (NCT04437368) as it did not reach the endpoints.

AAV-HGS CD59 developed by Hemera Biosciences/Janssen Research & Development (Johnson & Johnson). The drug is developed for the treatment of GA. This approach utilizes an adeno-associated virus (AAV) vector to deliver a soluble form of the TCC inhibitor CD59, directly into the eye via intravitreal injection. The therapeutic goal was to specifically block the terminal phase of the complement cascade on retinal cells and tissue, thereby mitigating complement-mediated damage in GA. However the clinical trial phase II (NCT05811351) was stopped before recruitment of patients because of missing functional activity of the protein.

CLG561 is developed by Alcon/Novartis to treat GA. CLG561 is a humanized Fab fragment derived from a monoclonal antibody targeting properdin, a key stabilizer and positive regulator of the AP C3 convertase. Administered via intravitreal injection, CLG561 aims to neutralize properdin and thereby inhibit AP activation. Phase I trials demonstrated that CLG561 was safe and well tolerated. In Phase II studies, the compound was tested both as monotherapy and in combination with the C5-targeting monoclonal antibody LFG316 (see below). Over a 12-month study period, neither the monotherapy nor the combination therapy significantly slowed GA lesion progression.

LFG316 (tesidolumab) developed by Novartis Pharmaceuticals is a humanized IgG1 monoclonal antibody targeting C5, of the terminal complement pathway. Phase I trials confirmed its safety and tolerability. However, in Phase II trials, monthly intravitreal injections of LFG316, while well tolerated, did not result in a statistically significant reduction in GA lesion growth.

### 5.7 Complement inhibition in AMD therapy: insights from clinical trials

A diverse array of complement inhibitors is currently under investigation for complement-mediated diseases, including AMD. These agents vary in structure and mechanism, targeting distinct components and pathways of the complement cascade. They include small molecules, mAbs, recombinant proteins, nanobodies, RNA interference agents, and gene therapy vectors (e.g., AAV).

Due to their varied physicochemical properties, these compounds require different routes of administration, including intravitreal, subretinal, intravenous (iv), subcutaneous, and oral delivery. Systemic administration (e.g., iv or oral) raises concerns about drug accessibility to retinal targets, especially when Bruch’s membrane remains intact or is only mildly compromised. Given the immune-privileged status of the retina, larger molecules such as mAbs may not effectively penetrate the intact retina via systemic routes, limiting their therapeutic potential in AMD. Phase II trials involving intravitreal C5-targeting mAbs suggest that terminal pathway inhibition alone may be insufficient to meet primary endpoints. This notion, however, is challenged by the clinical efficacy of the approved C5-targeting aptamer avacincaptad pegol, which highlights key differences between aptamer- and mAb-based therapies. These differences —such as drug type, molecule size, route of administration, biodistribution, and stability— appear to be critical factors in achieving lesion reduction in GA.

Two Factor D-targeting compounds, danicopan (NCT05019521) and lampalizumab (NCT02247479) were evaluated in clinical trials for GA. However, oral or intravitreal administration of these agents failed to demonstrate significant therapeutic benefit, leading to the discontinuation of both programs. Similarly, the C5 inhibitor eculizumab (NCT00935883) failed to significantly impact lesion growth in GA after 52 weeks of treatment. For CNV several anti-VEGF agents, aflibercept, bevacizumab, brolucizumab, faricimab, and ranibizumab have been approved and significantly improved clinical outcomes. These mAbs, administered via intravitreal injection, inhibit the growth and leakage of pathological neovascularization (128, 129).

## 6 Conclusion and outlook

The complement system has emerged as a central player in the pathogenesis of AMD, driving chronic inflammation and tissue injury at the RPE–BM–choriocapillaris interface. Complement inhibition has proven to be both biologically and clinically feasible as a therapeutic strategy, however, the clinical benefit observed so far remains modest, primarily slowing the progression of GA, rather than restoring vision. In addition, many of the currently available inhibitors require repeated intravitreal injections, which imposes a considerable treatment burden on patients and may affect long-term adherence. Several inhibitors tested in clinical trials have been unsuccessful and clinical outcomes across different complement–targeting approaches have been somewhat inconsistent. Suggesting that the optimal therapeutic target within the cascade remains to be defined. Gene-therapy-based strategies carry their own risks, since once the vector has been delivered and gene expression established, the intervention is difficult to reverse or adjust should adverse effects occur.

Despite strong genetic and biochemical evidence implicating dysregulation of the complement cascade in AMD, several key questions remain about how, where, and when the complement cascade should be targeted to achieve durable therapeutic benefit without compromising physiological immune defense. A major consideration is the level at which complement should be targeted. Inhibition at the level of C3 provides broad blockade of the cascade, potentially preventing all downstream inflammatory and lytic effects, but carries a higher risk of immunosuppression. In contrast, distal inhibition at the level of C5 or the TCC has not been that successful so far. Determining the optimal level of intervention may depend also on disease stage and patient genotype.

Closely related is the question of which inflammatory pathways of complement should be modulated. While the alternative pathway is most strongly implicated in AMD pathogenesis, cross-talk with the classical lectin and intracellular pathways complicates selective targeting. Modulating the amplification loop of the alternative pathway, rather than complete suppression, might provide long-term protection while maintaining basal immune competence.

The choice of therapeutic modality is equally critical. Monoclonal antibodies and recombinant proteins offer high specificity but require repeated intravitreal injections, raising the risk of local complications. Smaller proteins, aptamers, nanobodies, or RNA-based therapeutics (such as siRNA or gene therapy vectors) may allow more sustained effects through improved tissue penetration or endogenous production of inhibitory molecules. Gene therapy approaches targeting complement regulators are promising; however, they must be carefully balanced and may cause more unwanted side effects.

The route of administration should be matched to both the pharmacologic properties of the compound and the intended site of action. Intravitreal delivery remains the gold standard for achieving therapeutic concentrations at the RPE–choroid interface, whereas subretinal administration may be more suitable for gene vector–based therapies that require transduction of RPE cells. Systemic (intravenous or subcutaneous) routes could be considered for complement inhibitors with ocular tropism or when systemic complement dysregulation contributes to disease progression. Oral administration, while convenient, remains challenging due to bioavailability and the need for ocular-specific targeting.

An important conceptual distinction lies between complement inhibitors that suppress activity and complement regulators that restore homeostasis. Agents that mimic or enhance endogenous regulatory proteins, such as Factor H, might achieve therapeutic benefit with fewer side effects, preserving the delicate balance between immune surveillance and inflammation. This raises the possibility that combination strategies, pairing a regulator with a targeted inhibitor or designing dual-action molecules, could deliver synergistic effects—offering both immediate control of excessive activation and sustained restoration of physiological complement balance.

Equally crucial are the timing and duration of therapy. Early intervention (prior to irreversible RPE and photoreceptor loss) may offer the greatest potential for structural and functional preservation. However, defining when to initiate and how long to maintain treatment requires longitudinal studies correlating complement activity biomarkers with disease progression and therapeutic response.

Finally, it remains to be determined whether complement therapy can directly influence RPE physiology, potentially replacing or restoring atrophic areas, enhancing RPE fluidity, and promoting cellular remodeling. Emerging evidence suggests that complement modulation not only dampens inflammation but may also foster a microenvironment conducive to RPE regeneration and re-establishment of retinal homeostasis.

In summary, the successful translation of complement-targeted therapy for AMD will require an integrated approach, optimizing the level and mode of complement modulation, matching the therapeutic modality to the route of administration, and tailoring timing and duration to disease stage. By balancing inhibition and regulation, next-generation therapies may move beyond halting degeneration to actively promoting retinal repair and long-term visual preservation.
